# Patient Safety Events Among People from Ethnic Minority Backgrounds: A Retrospective Medical Record Review of Australian Cancer Services

**DOI:** 10.1007/s40615-025-02318-8

**Published:** 2025-02-27

**Authors:** Ashfaq Chauhan, Kathryn Joseph, Melvin Chin, Meron Pitcher, Carlene Wilson, Elizabeth Manias, Guncag Ozavci, Hui Gan, Bronwyn Newman, Ramesh Lahiru Walpola, Holly Seale, Ramya Walsan, Reema Harrison

**Affiliations:** 1 Centre for Health Systems and Safety Research, Australian Institute of Health Innovation, Macquarie University, Level 6, 75 Talavera Road, Macquarie Park, Sydney, NSW 2109 Australia; 2 Medical Oncology, Prince of Wales Hospital, South Eastern Sydney Local Health District, Randwick, NSW Australia; 3 General & Breast Surgery Unit, Western Health, Footscray, VIC Australia; 4 Olivia Newton-John Cancer Wellness and Research Centre, Austin Hospital, Heidelberg, VIC Australia; 5 School of Psychology & Public Health, La Trobe University, Bundoora, VIC Australia; 6 School of Nursing and Midwifery, Monash University, Victoria, Australia; 7 Centre for Quality and Patient Safety Research, School of Nursing & Midwifery, Institute for Health Transformation, Deakin University and Alfred Health, Melbourne, VIC Australia; 8 Medical Oncology, Austin Hospital, Heidelberg, VIC Australia; 9 School of Cancer Medicine, La Trobe University, Bundoora, VIC Australia; 10 Department of Medicine, University of Melbourne, Victoria, Australia; 11 School of Health Sciences, UNSW Sydney, Kensington, NSW Australia; 12 School of Population Health, UNSW Sydney, Kensington, NSW Australia; 13 Centre for Epidemiology and Biostatistics, Melbourne School of Population and Global Health, The University of Melbourne, Melbourne, VIC Australia

**Keywords:** Ethnic minorities, Patient safety, Medication safety, Health services research, Cancer

## Abstract

**Objectives:**

People from ethnic minority backgrounds are exposed to greater risk of patient safety events (such as healthcare-acquired infections and medication errors) occurring in their healthcare. However, evidence of the type and frequency of patient safety events occurring in cancer care among patients from ethnic minority background is lacking. This study sought to address this evidence gap.

**Design:**

A two-stage retrospective medical record review was conducted at four cancer services in two Australian states. Eligible medical records at each service that were identified as belonging to ethnic minority patients were reviewed by two clinician researchers in stage one, followed by authentication of extracted data by a site-specific cancer clinician in stage two. Descriptive statistics were used to report the frequency and type of safety events. Chi-square and independent sample *T*-tests were used to examine the association between safety events and patient socio-cultural indicators.

**Results:**

A total of 628 patient records were included. Of the 628 patient records, 212 (33.75%) documented at least one safety event. A total of 410 safety events were documented in the 212 patient records. Medication-related safety events were most commonly documented (121/410, 29.5%), followed by clinical process/procedure-related safety events (76/410, 18.5%) and patient accidents (60/410, 14.6%). The occurrence of a safety event was associated with patient records that documented ‘no interpreter was required’.

**Conclusion:**

Patient safety events in cancer care occur frequently among patients from ethnic minority backgrounds. Unsafe cancer care for this population is associated with inadequate use of interpreters, lack of shared understanding and expectations of care processes linked to cultural and linguistic barriers. Approaches to enhance engagement are required.

**Supplementary Information:**

The online version contains supplementary material available at 10.1007/s40615-025-02318-8.

## Introduction

A patient safety event describes a healthcare event that could have or did result in patient harm due to the care provided or omitted rather than the disease [1]. Common patient safety events include healthcare-acquired infections, medication errors and the lack of adherence to policy and procedures when delivering care [1]. Studies examining the rate of patient safety events among the general population have consistently indicated that at least one in ten patients accessing hospital care is exposed to a patient safety event [2–9].

People from ethnic minority backgrounds include those being born overseas, who have one or more parents born overseas, who speak a non-mainstream language, practice a religion, culture or hold a faith beyond the national, mainstream religion, culture or faiths [10, 11]. Systematic review evidence demonstrates that people from ethnic minority backgrounds worldwide experience higher rates of patient safety events, coupled with poorer clinical outcomes, across a range of health settings when compared with those from a non-ethnic minority background [12, 13]. Minimising differences in exposure to unsafe care is central to achieving equitable healthcare quality, which is a priority in contemporary health system and service frameworks [14–17].

Evidence of the association between safety events and ethnic diversity has largely originated from the United States (US). Many service and population-based studies demonstrate that people from ethnic minority groups are at higher risk of safety events such as healthcare-acquired infections and medication errors [12, 18, 19]. Ethnicity has been predominantly defined by race in these studies, with comparisons made between Black, Caucasian, Asia/Pacific Islanders and Hispanic populations. Increased incidence of patient safety events is noted among patients with low-English or national language proficiency [20] with studies indicating safer health outcomes when trained professional interpreters are used [12]. Retrospective review studies that have explored language barriers alone show increased incidence of patient harm among low-English proficiency patients [20] and longer hospital stays, accompanied with increased odds of a serious or sentinel event among Spanish speaking children [21].

Beyond the US, studies that report disparate patient safety outcomes among ethnic minority populations have done so with regard to a single patient safety issue, e.g. medication safety [13, 22] or health setting, e.g. obstetrics and neonatal care, and often are single-site studies [23, 24]. Exploratory qualitative research has also been conducted providing critical insight into key factors influencing safe care among this population, which suggests that national language proficiency, health literacy, differences in perception of illness and understanding of care expectations between patients from ethnic minority backgrounds and their clinicians and the extent of family involvement may impact patient safety outcomes for patients from ethnic minority backgrounds [25–27].

The potential patient safety events are particularly high in cancer care [28]. A retrospective record review of 6720 medical records of general population patients identified that 24.2% (197/812) of the hospital admissions for cancer patients had a safety event compared to 17.4% (1027/5908) of admissions of other patients at one hospital in Norway [29]. Another study that included adverse drug reactions and drug toxicities in their definition of patient safety events documented that approximately 34% (136/400) of patients accessing a cancer service in the United States experienced a safety event over a 12-month follow-up period (30). Despite the established evidence that the patients from ethnic minority backgrounds are exposed to greater risk of patient safety event and that there is a high risk of safety events occurring in cancer care, there is a lack of evidence of the nature and rate of patient safety events among patients from ethnic minority backgrounds in cancer care [12, 31]. This study aimed to fill in this gap by providing evidence on the frequency and nature of patient safety events among patients from ethnic minority backgrounds accessing caner care. The study also aimed to examine any association between patient safety events and routinely collected socio-cultural characteristics of ethnic minority patients in the medical records.

## Method

### Study Design

A two-stage retrospective medical record review was conducted [30, 32–34].

### Ethics

Ethics approval was received from two National Health and Medical Research Council accredited Human Research Ethics Committees (Reference numbers: 2019/ETH12997 and HREC/60908). A waiver of consent was approved for this retrospective medical record review.

### Setting

The study was conducted across four cancer services located in four distinct government administrative areas in Australia [two in the state of New South Wales (NSW) and two in the state of Victoria (VIC)]. Both states have a large and diverse ethnic minority population [35]. All four cancer services were based within publicly funded, teaching hospitals that ranged from 450 to 670 beds. Each service provided comprehensive cancer care including chemotherapy, radiotherapy, surgical and palliative care across inpatient and outpatient (including community) settings.

### Sample

The minimum sample size required to determine the prevalence of a safety event was 300 medical records of patients from ethnic minority backgrounds with cancer, based on estimated rate of 25% and 5% margin of error [36]. Given the inclusion of four distinct sites across two states, we aimed to review 600 medical records and 300 patient records per state.

### Eligibility

Medical records were eligible for inclusion if they (a) had one or more of the following Australian Bureau of Statistics (ABS) ethnic minority indicators present (country of birth, language spoken at home, preferred language or interpreter required) [11]; (b) had a diagnosis of cancer, of any kind and documented; (c) had been seen by a health professional at the participating cancer service for a cancer-related illness and/or management for the first time between 1 January 2018 and 31 December 2018 (first episode of care) and (d) belonged to a patient 18 years of age or older.

Known common complications of cancer treatment such as febrile neutropenia, peripheral neuropathy, bone marrow suppression and other adverse drug reactions that could occur due to drug toxicities were excluded because these events may occur even when appropriate processes of care were followed [28, 37]. This criterion was applied with the help of the site-specific cancer clinician. For consistency, patients’ records were reviewed for the care they received over a 12-month period following the date of the first episode of care at the participating cancer service. Records from 2018 to 2019 were selected in order to provide study data that was not impacted by the COVID-19 pandemic period.

### Data Collection Instruments

A validated oncology trigger tool [30], developed in the United States, was used in this study (Supplementary File 1). This trigger tool included 76 distinct triggers grouped in six categories (laboratory, general care, vital signs, orders, consults and medication related) and included triggers such as hospital readmission within 72 h of discharge or ambulatory surgery. The corresponding laboratory values, units and drug names were adapted to Australian context with assistance of a medical oncologist (MC) prior to its use (Supplementary File 1). A data collection tool (Supplementary File 2) was also developed in collaboration with all authors and members of the research project team to collect data relevant to the study objectives in a standardised manner [33].

### Data Sources

All four cancer services had different electronic medical record (EMR) systems in place across inpatient and outpatient settings (Supplementary File 3). Data sources consisted of the notes made electronically and/or scanned paper-based documents uploaded as Portable Document Format (PDF) on the EMR systems. One cancer service in VIC had transitioned to a different EMR system in 2018–2019. At this service, the previous system exclusively included paper records uploaded as PDFs while the new system was similar to the other three services in which notes could be entered electronically along with possibility of uploading scanned documents. Data sources comprised of all progress and other clinical notes, medical and clinical assessments and reviews, doctors’ correspondence, laboratory notes and results, and family conferences and correspondence notes.

### Project Team and Reviewers’ Training

A project team was established and consisted of a project lead (RH), a lead clinician researcher (AC), two clinical research fellows (KJ, GO), two research assistants (COD, ACh), four site-specific lead cancer clinicians (MC, MP, HG, HC) and other research team members (BN, HS, RLW, EM, CW, RW). Training was provided to five members of the team who collected data (AC, KJ, GO, COD, ACh) before the commencement of data collection by the research project lead (RH). A standardised process for data collection, considering the constraints of each site, was established. The process involved an initial meeting between project lead, lead clinician researcher and other site-specific clinician researcher/research assistant and site-specific lead cancer clinician. This initial meeting assisted researchers collecting data to familiarise themselves with the EMR systems used at the respective service, address logistical requirements before commencing data collection, agree on the schedule for data collection and establish a procedure for communication to clarify any queries.

### Identification of Medical Records

A list of patients admitted during the eligible timeframe was compiled by the data administration departments at each service with the assistance of site-specific lead cancer clinician. Given the limitations of socio-cultural data captured in medical record review systems, manual identification of ethnic minority patient records was required. This process was completed by two reviewers (AC and KJ). To manage this process, an online random number generator was used. A batch of 50 random numbers was generated using the online random number generator. These numbers were then applied to the list of patients compiled by the data administration department. The eligibility of the corresponding patient records was checked and those eligible were included for review. Once the batch of 50 random numbers was exhausted, another batch of 50 random numbers was generated and applied to the patient list to identify eligible records. This process was repeated until the required number of medical records were identified and included for review at each cancer service. After identification, each patient record was reviewed for a follow-up period of 12 months from the date of their first episode of care.

### Data Extraction

The data extraction method was piloted with 20 medical records at one site in NSW to test the feasibility of the data extraction tool, ensure consistency in data extraction processes between reviewers and make any necessary changes before implementing the tool across the four services. The process of data extraction was standardised across the four services and consistency was established by comparing data extraction between two clinician reviewers who both examined 10% of the medical records at each service. This allowed reviewers to establish a common understanding of the type of data to be collected, compare their findings and discuss and resolve any differences with the help of the project lead. After this, each respective clinician independently conducted record reviews. A weekly meeting occurred between the two clinician reviewers and the project lead during data extraction to discuss the findings and raise any issues experienced, which were then collectively resolved. The process was followed until all the patient records were reviewed.

In stage 1, patient characteristics were extracted for each record using the data extraction tool. Following this, the oncology trigger tool was applied and the records were reviewed for the presence of any trigger. If no trigger was identified, no further data were extracted. If a trigger was present, the medical record was subject to a full review to identify if any safety events occurred during the 12-month follow-up period from the date of first episode of care. When a safety event was identified, the notes were examined to identify and collect information relating to the safety event corresponding to study objectives.

In stage 2, the safety events identified through data collection by the research clinicians were reviewed by the respective lead cancer clinician at each service. This was an essential step to validate the safety events and enhance the rigour of the study. Each lead cancer clinician examined the data collected in the data extraction tool with the clinician researcher from each site. This review process either occurred in batches throughout the process or after all the data were extracted at each service based on clinician’s preference and availability. Each safety event was reviewed, and a decision was made by the lead cancer clinician as to whether the event was determined as a safety event based on their clinical knowledge and familiarity with the care processes at the respective site. Any discrepancies observed in determination of safety events by clinician researchers and lead cancer clinicians were resolved by discussion or extracting further information from the records as needed to reach a conclusion.

### Safety Events Classification

At the time of data extraction, each safety event was assigned a safety event classification based on the World Health Organisation (WHO) International Classification of Patient Safety (ICPS) framework [1, 38, 39]. To ensure consistency, each event was classified to the category that led most directly to the identified patient harm or potential for patient harm [39]. In stage two, the ICPS framework was independently applied by three patient safety researchers (RH, RLW and AC) to validate the classifications. Any disputes were resolved through discussion.

### Data Analysis

Data were analysed using SPSS software (IBM SPSS Statistics for Windows, Version 29.0. Armonk, NY: IBM Corp). Data from the four services were aggregated and collectively analysed to report the association between patient characteristics and safety events. The nature and rate of safety events were reported collectively and for each health service. Data were categorised and frequencies and proportions were calculated for indicators relating to the study objectives.

Pearson’s chi-square test of independence was conducted to determine differences between the four services for patient characteristics and the occurrence of safety events, and to determine any association between the independent indicators (i.e. demographic characteristics of the patients) and the occurrence of any safety event, types of safety events and number of safety events. Missing values were coded as ‘system missing’ and were not included in the analysis. As there was no reference group for ‘Regional classification for country of birth’, no association was examined between this variable and the occurrence, number or type of safety events. An independent *T*-test was conducted to examine statistically significant differences between the number of safety events as continuous variable and the interpreter required status (recorded as ‘yes’ or ‘no’) as well as the preferred language status (recorded as ‘English’ and ‘non-English).

### Patient and Public Involvement

The Consumer Advisory Group (CAG) was developed as part of this project. The CAG consisted of six members who were from ethnic minority backgrounds and had lived experience of cancer. The CAG provided feedback on the data extraction tool and met with the project team through regular meetings to discuss the methodology and emerging findings.

## Results

**Fig. 1:**
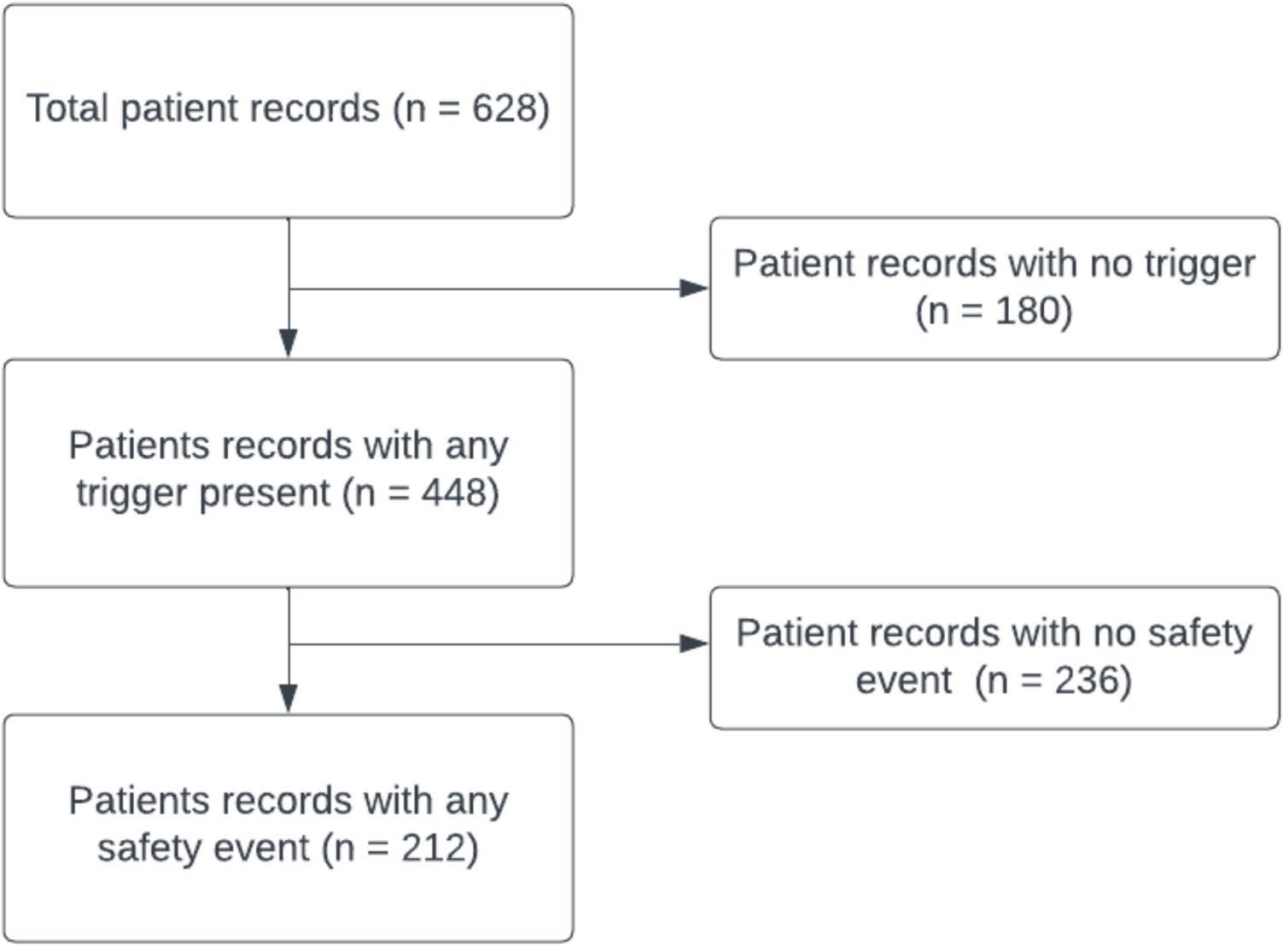
Flowchart describing the identification of number of patient records with any safety event

A total of 628 patient records were reviewed (Fig. 1). Over one-third of the patient records (212/628 33.75%) recorded at least one safety event. A total of 410 safety events were documented across 212 patient records.

Table 1 describes the demographics and socio-cultural characteristics documented in the patient records reviewed across the four cancer services. Over half of the records (336/628; 53.5%) were for male patients. The median age was 69 years (SD 13.39) and the age range was 21–99 years. For socio-cultural indicators, statistically significant differences were observed in between the four cancer services for ‘regional classification for country of birth’ and ‘preferred language’ confirming the diversity of ethnic minority groups accessing the four sites. No significant difference was observed between the sites for the ‘interpreter required’ category. For clinical indicators, statistically significant differences were observed in between the four cancer services for ‘cancer type’ and ‘primary treatment received’ categories.

**Table 1:** Demographics and socio-cultural characteristics of the extracted patient records and how these differed between hospital collection sites

Variable	Service A (*N* = 150)	Service B (*N* = 150)	Service C (*N* = 228)	Service D (*N* = 100)	Total (*N* = 628)	*χ*^2^ (*p*, *n*)
***Sex***						6.9 (0.075, 628)
Male	83 (55.3%)	78 (52%)	117 (51.3%)	64 (64%)	336 (53.5%)	
Female	67 (44.7%)	72 (48%)	111 (48.7%)	36 (36%)	292 (46.5%)	
***Age***						5.3 (0.804, 628)
19–45 years	10 (6.7%)	13 (8.7%)	19 (8.3%)	6 (6%)	48 (7.6%)	
46–60 years	26 (17.3%)	34 (22.7%)	48 (21%)	16 (16%)	124 (19.7%)	
61–75 years	66 (44%)	67 (44.7%)	102 (44.8%)	48 (48%)	283 (45.1%)	
76 and over	48 (32%)	36 (24%)	59 (25.9%)	30 (30%)	173 (27.6%)	
***Region of country of birth***						112.9 (< 0.001, 628)
Southern Europe	35 (23.3%)	25 (16.7%)	106 (46.5%)	47 (47%)	213 (34%)	
South-Eastern Asia	25 (16.7%)	30 (20%)	24 (10.5%)	20 (20%)	99 (15.8%)	
Eastern Asia	29 (19.3%)	11 (7.3%)	16 (7%)	3 (3%)	59 (9.4%)	
Southern Asia	3 (2%)	27 (18%)	23 (10%)	4 (4%)	57 (9%)	
Western Asia	10 (7.3%)	15 (10%)	24 (10.5%)	4 (4%)	53 (8.4%)	
Eastern Europe	17 (11.3%)	5 (3.3%)	10 (4.5%)	5 (5%)	37 (5.9%)	
Other*	31 (20.7%)	37 (24.7%)	25 (11%)	17 (17%)	110 (17.5%)	
***Preferred language***						50.8 (< 0.001, 624)
Non-English	104 (69.3%)	57 (38%)	82 (36%)	35 (35%)	278 (44.3%)	
English	45 (30%)	92 (61.3%)	144 (63.1%)	65 (65%)	346 (55%)	
Not recorded	1 (0.7%)	1 (0.7%)	2 (0.9%)	-	4 (0.7%)	
***Interpreter required***						7 (0.066, 617)
Yes	53 (35.3%)	35 (23.3%)	58 (25.4%)	31 (31%)	177 (28.2%)	
No	97 (64.7%)	115 (76.7%)	166 (72.8%)	62 (62%)	440 (70%)	
Not recorded	-	-	4 (1.8%)	7 (7%)	11 (1.8%)	
***Cancer type***						72.5 (< 0.001, 628)
Gastrointestinal	37 (24.7%)	36 (24%)	38 (16.7%)	35 (35%)	146 (23.1%)	
Breast	22 (14.7%)	41 (27.3%)	46 (20.1%)	14 (14%)	123 (19.5%)	
Lung	15 (15%)	33 (22%)	35 (15.3%)	14 (14%)	97 (15.4%)	
Haematological	19 (12.7%)	11 (7.3%)	50 (21.9%)	7 (7%)	87 (13.9%)	
Prostate	17 (11.3%)	11 (7.3%)	9 (4%)	14 (14%)	51 (8.1%)	
Genital	11 (7.3%)	6 (4%)	9 (4%)	1 (1%)	27 (4.3%)	
Urological	4 (2.7%)	1 (0.7%)	9 (4%)	5 (5%)	19 (3%)	
Head and neck	11 (7.3%)	1 (0.7%)	2 (0.9%)	-	14 (2.2%)	
Others	14 (9.3%)	10 (7.7%)	30 (13.1%)	10 (10%)	64 (10.2%)	
***Primary treatment received***						62.9 (< 0.001, 628)
Combination*	49 (32.7%)	66 (44%)	70 (30.7%)	35 (35%)	220 (35%)	
Chemotherapy	15 (10%)	28 (18.7%)	45 (19.7%)	27 (27%)	115 (18.4%)	
Surgery	19 (12.7%)	9 (6%)	25 (11%)	12 (12%)	65 (10.4%)	
Radiotherapy	32 (21.3%)	19 (12.6%)	7 (3%)	10 (10%)	68 (10.7%)	
Other	35 (23.3%)	28 (18.7%)	81 (35.5%)	16 (16%)	160 (25.5%)	

Column percentages indicate the proportionate prevalence of each variable level within each health service. Percentages are not reported for cells with zero counts. *p* values (< 0.05) refer to the statistical significance of chi-squared tests when comparing each demographic and clinical variable between the four health services. *Combination indicates any combination of surgery, chemotherapy or radiotherapy as primary treatment recorded in the patient record. *Other for regions of country of birth includes Caribbean, Central America, Central Asia, Eastern Africa, Melanesia, Northern Africa, Northern Europe, Polynesia, South America, Southern Africa, Western Africa and Western Europe

Large variations were recorded for socio-cultural indicators across the four services. Collectively, over 90 different countries of birth were identified, with Greece (72/628; 11.5%), Italy (53/628; 8.4%) and China (47/628; 7.5%) representing the top three countries of birth documented in patient records. A total of 48 non-English languages were recorded as the preferred language, along with English, in the patient records. Due to these large variations, country of birth data was categorised into regional geographical areas [40] and language-related data were categorised into two categories (English and non-English). For one cancer service (service B), a discrepancy was observed in the administrative data collected for the ‘interpreter required’ indicator whereby 26/150 records had conflicting information recorded between the inpatient and outpatient EMR systems. For consistency, this information was recorded as ‘interpreter required’. Notably, three services did not routinely collect data for the ‘Language spoken at home’ indicator in the absence of a dedicated EMR field. This variable was not subject to further analysis and reporting.

### The Frequency of Safety Events

Table 2 describes the socio-cultural characteristics documented in the patient records with any safety event present reviewed across the four cancer services. For the two socio-cultural indicators, ‘preferred language’ and ‘interpreter required’, statistically significant difference was observed in between cancer services for patients records with any safety event. Service A recorded more safety events among patient records that had documented that an interpreter was required, and the preferred language was non-English when compared to other three services that recorded more safety events in records that had documented that an interpreter was not required and the preferred language as English.

**Table 2:** Demographic and socio-cultural characteristics of the extracted patient records for which there was at least one safety event and how these differed between sites

Variable	Service A (*N* = 47)	Service B (*N* = 57)	Service C (*N* = 83)	Service D (*N* = 25)	Total (*N* = 212)	*χ*^2^ (*p*, *n*)
***Sex***						0.27 (0.964, 212)
Male	28 (59.6%)	33 (57.9%)	50 (60.2%)	16 (64%)	127 (59.9%)	
Female	19 (40.4%)	24 (42.1%)	33 (39.8%)	9 (36%)	85 (40.1%)	
***Age***						8 (0.205, 212)
19–60 years	7 (14.9%)	16 (28.1%)	19 (22.9%)	2 (8%)	44 (20.7%)	
61–75 years	21 (44.7%)	28 (49.1%)	39 (47%)	16 (64%)	104 (49.1%)	
76 and over	19 (40.4%)	13 (22.8%)	25 (30.1%)	7 (28%)	64 (30.2%)	
***Preferred language***						23.9 (< 0.001, 211)
English	10 (21.3%)	32 (56.1%)	52 (62.7%)	16 (64%)	110 (51.9%)	
Non-English	37 (78.7%)	25 (43.9%)	30 (36.1%)	9 (36%)	101 (47.6%)	
Not recorded	-	-	1 (1.2%)	-	1 (0.5%)	
***Interpreter required***						11.8 (0.008, 210)
No	23 (49%)	37 (65%)	65 (78.3%)	15 (60%)	140 (66%)	
Yes	24 (51%)	20 (35%)	18 (21.7%)	8 (32%)	70 (33%)	
Not recorded	-	-	-	2 (8%)	2 (1%)	
***Cancer type***						29.9 (0.008, 212)
Gastrointestinal	18 (38.3%)	18 (31.6%)	15 (18.1%)	10 (40%)	61 (28.8%)	
Lung	7 (14.9%)	14 (24.6%)	19 (22.9%)	5 (20%)	45 (21.2%)	
Haematological	3 (6.4%)	3 (5.2%)	21 (25.3%)	2 (8%)	29 (13.7%)	
Breast	4 (8.5%)	11 (19.3%)	7 (8.4%)	2 (8%)	24 (11.3%)	
Others*	15 (31.9%)	11 (19.3%)	21 (25.3%)	6 (24%)	53 (25%)	
***Primary treatment received***						5.3 (0.806, 212)
Combination*	25 (53.1%)	28 (49.1%)	33 (39.8%)	13 (52%)	99 (46.8%)	
Chemotherapy	6 (12.8%)	10 (17.6%)	21 (25.3%)	4 (16%)	41 (19.3%)	
Surgery	7 (14.9%)	6 (10.5%)	12 (14.5%)	3 (12%)	28 (13.2%)	
Other*	9 (19.2%)	13 (22.8%)	17 (20.4%)	5 (20%)	44 (20.7%)	
***Number of safety events per record***						8.7 (0.175, 212)
1 safety event	31 (66%)	37 (64.9%)	37 (44.6%)	16 (64%)	121 (57.1%)	
2 safety events	8 (17%)	11 (19.3%)	24 (28.9%)	4 (16%)	47 (22.2%)	
≥ 3 safety events	8 (17%)	9 (15.8%)	22 (26.5%)	5 (20%)	44 (20.7%)	

Column percentages indicate the prevalence of each variable level within each health service. Percentages are not reported for cells with zero counts. *p* values (< 0.05) refer to the statistical significance of differences between the health services as calculated through chi-squared tests. *Other cancer type includes prostate, head and neck, sarcomas, genital, urological, hepatic and other cancers. The ‘regional classification for country of birth’ was not calculated due to smaller sub-groups. Combination indicates any combination of surgery, chemotherapy or radiotherapy as primary treatment recorded in the patient record

A chi-square test for independence, after collapsing all health service level data together, indicated significant association between occurrence of safety event with sex (*χ*^2^ = 5.2, *p* = 0.022, *n* = 628; safety events recorded more among male), cancer type (*χ*^2^ = 27.4, *p* < 0.001, *n* = 628; safety events recorded more in patients with gastrointestinal cancer) and primary treatment type received (*χ*^2^ = 28.7, *p* < 0.001, *n* = 628; safety events recorded more among those who received combination of treatment). The test also indicated a trend between the occurrence of safety event and age range (*χ*^2^ = 7.7, *p* = 0.051, *n* = 628; safety events recorded more among patients in the age range of 61–75 years). The test also indicated trend between occurrence of safety event and the interpreter requirement documentation (*χ*^2^ = 3.4, *p* = 0.067, *n* = 617; safety events were recorded more when interpreter was documented as not required).

The independent sample *T*-test indicated no significant difference in the number of safety events identified in patient records that recorded English as a preferred language (M = 0.60, SD = 1.33) and those that recorded non-English language as a preferred language (M = 0.73, SD = 1.37); *t* (622) =  − 1.243, *p* = 0.16) as administrative data. Similarly, no significant difference in the number of safety events was identified in patient records that recorded interpreter was not required (M = 0.65, SD = 1.45) and those that recorded an interpreter was required (M = 0.68, SD = 1.083); *t* (617) =  − 0.298, *p* = 0.54) as administrative data.

### Type of Safety Events

A range of safety event types were recorded across inpatient, outpatient and ED settings (Table 3). More than two-thirds of the safety events (298/410, 72.7%) documented were related to inpatient care rather than outpatient (97/410, 22.4%) or emergency care (15/410, 5.6%). Safety events related to inpatient care were recorded more frequently in all four services.

**Table 3:** Nature and type of safety events at each site and overall

Variable	Service A (*N* = 73)	Service B (*N* = 88)	Service C (*N* = 203)	Service D (*N* = 46)	Total (*N* = 410)
***Setting***					
Inpatient	48 (65.8%)	56 (63.6%)	163 (80.3%)	31 (67.4%)	298 (72.7%)
Outpatient	22 (30.1%)	25 (28.5%)	37 (18.2%)	13 (28.3%)	97 (23.7%)
ED	3 (4.1%)	7 (7.9%)	3 (1.5%)	2 (4.3%)	15 (3.6%)
***Type (as per WHO ICPS taxonomy)***					
Medication/IV fluids	22 (30.1%)	32 (36.4%)	58 (28.6%)	9 (19.6%)	121 (29.5%)
Clinical process/procedure	10 (13.7%)	20 (22.7%)	39 (19.2%)	7 (15.2%)	76 (18.5%)
Patient accidents	15 (20.5%)	10 (11.4%)	25 (12.3%)	10 (21.7%)	60 (14.6%)
Medical device/equipment	5 (6.9%)	7 (8%)	19 (9.4%)	5 (10.9%)	36 (8.8%)
Healthcare-associated infection	6 (8.2%)	7 (8%)	21 (10.3%)	1 (2.2%)	35 (8.5%)
Behaviour	4 (5.5%)	4 (4.5%)	16 (7.9%)	6 (13%)	30 (7.3%)
Documentation	3 (4.1%)	4 (4.5%)	14 (6.9%)	2 (4.4%)	23 (5.6%)
Other*	8 (11%)	4 (4.5%)	11 (5.4%)	3 (13%)	29 (7.2%)

Column percentages indicate the proportionate prevalence of each variable level within each health service. Percentages are not reported for cells with zero counts. *Other includes clinical administration, blood and blood products, oxygen/gas/vapour and resource/management safety event type. Association between type of safety events and setting was not examined due to smaller sub-groups

Safety events linked to medication/IV fluid intake were documented most frequently (121/410, 29.5%) in both outpatient (36/97, 37.1%) and inpatient (79/298, 26.5%) setting, but were reported in a higher proportion of records in outpatient settings. These types of safety events ranged from problems with dose charting (e.g. insulin not charted, non-cancer medication not charted, wrong dose of chemotherapy calculated), issues with drug administration (e.g. antibiotics not given as charted, frusemide given incorrectly in the morning, incorrect administration of moxonidine, missed medication) and patient errors in medication dose intake (e.g. patient stopped taking blood pressure medication, patient took the wrong dose of dexamethasone, patient took the wrong dose of oxycontin, patient started metoprolol medication on his own). For inpatient care, medication-related safety events were largely related to medication administration and wrong prescription. For outpatient care, these events were largely related to taking the wrong dose or medication and non-adherence to medication guidance.

Safety events related to clinical processes and procedure (76/410, 18.5%) also occurred in inpatient (53/298, 17.8%) and outpatient settings (17/97, 17.5%), resulting in pressure ulcers and inappropriate care delivery (e.g. wrong instructions for drain removal following breast surgery). Patient accidents largely comprised falls and occurred mostly in inpatient settings (58/60, 96.5%). Medical device and equipment-related safety events occurred mostly in inpatient settings (30/36, 83.3%) and consisted of dislodgment, blockage or leakage of nasogastric tubes, chest drains or intravenous cannulas. Healthcare-associated infections (35/410, 8.5%) occurred both in inpatient care and outpatient care where patients developed infection, blister, abscess or pneumonia following a surgery or admission. Safety events related to patient behaviour (30/410, 7.3%) included taking medication brought from home after admission as an inpatient, refusal to take medication given by nurse due to confusion about the doctor’s guidance, non-compliance with conducting blood-tests prior to chemotherapy and intentionally removing catheter as they found it uncomfortable.

## Discussion

### Principle Results

The medical record review indicated that almost one-third of patients from ethnic minority backgrounds accessing cancer care had at least one safety event documented in the 12 months following their first episode of care. A range of safety event types were identified, but medication-related safety events were most frequently documented. Data for the indicator ‘language spoken at home’ used to identify ethnic minority status in hospital records were not routinely collected by all sites. Inconsistent interpreter requirements to assist with communication were documented in EMRs. A trend was observed for greater occurrence of safety events when interpreters were documented as not required.

### Comparison with Prior Work

To our knowledge, this was a first study exploring frequency and nature of safety events among people from ethnic minority backgrounds accessing cancer care. The rate of safety events among the general population accessing hospital care is widely accepted to be approximately 10% [41] and is estimated to be 24% among hospitalised cancer patients in one study [29]. Although we did not collect comparable data from non-ethnic minority patients in the present study, our findings that at least one in three patients from ethnic minority backgrounds accessing cancer care in the hospital was exposed to a safety event indicate that these patients are frequently at risk of unsafe care when considered in the context of existing studies. Although a retrospective medical record review study conducted in a cancer service to examine adverse events among the general population suggests a rate of 34% [30], the outcomes studied are not directly comparable.

A large proportion (almost one-third) of safety events identified in the present study were medication related. There is an increased emphasis on identification and reporting of medication-related safety events due to severe consequences associated with them, especially in the context of cancer care [42, 43]. This may have contributed to increased documentation of such events in patient records. Nonetheless, international studies outside of cancer care have also documented a higher incidence of medication-related safety events among patients from ethnic minority backgrounds [44, 45]. Increased incidence of medication-related safety events among patients from ethnic minority backgrounds is attributed to communication barriers due to cultural and linguistic differences [13, 27, 46]. A recent qualitative study exploring experiences of 54 Australian cancer service staff identified that resources (such as interpreters and translated information) to support communication during tasks such as routine chemotherapy treatment sessions was limited [46]. Further cultural and religious beliefs of cancer as a shameful disease and guarding of cancer diagnosis and treatment by family members compounded communication with some patients from ethnic minority backgrounds [46]. These specific communication factors may contribute to reduced shared understanding of medication instructions and care processes, potentially increasing the risk of patient harm among patients from ethnic minority backgrounds.

The study indicated a trend between documentation of a safety event occurring with administrative data indicating an interpreter was not required. Inconsistencies were observed in the patient medical records with some patients who used an interpreter having a record that indicated that the interpreter was not required. This indicates a disparity in language needs assessment. Assessing whether an interpreter is required is a significant issue for all health services, impacting equity in cancer care planning and delivery for patients from ethnic minority backgrounds. A retrospective review of 19,627 inpatient episodes of care at one hospital in Australia identified that for almost 16% (*n* = 3287) of episodes of care that identified need of interpreter, only 17% (*n* = 526) episodes of care received healthcare interpreter service [47]. The same review also noted that for 201 patients, the interpreter need was not identified at the time of the admission [47]. Further, reliability of the ethnic minority patient’s confidence in their own English language competence and practitioner’s assessment of English language proficiency are questioned (48, 49). A formal system for assessing competence, particularly for health literacy, may be required (e.g. Newest Vital Sign or the Test of Functional Health Literacy in Adults (TOFHLA)) [50, 51].

### Implications

Given that people with cancer are increasingly being managed in outpatient settings, the high proportion of medication-related safety events associated with outpatient care is a considerable concern [28]. Medication management plans are proposed by policy organisations internationally to enhance medication safety in outpatient settings by fostering patient engagement in their care [42, 43]. Direct translations of medication management plans to enhance patient education about medication management at home may not be suitable [52]. Specific strategies developed using co-design to suit the socio-cultural and language requirements of ethnic minority patients to support their meaningful engagement in medication management are needed [17, 53].

Documentation of patient safety events related to understanding of medication instructions or care process instructions indicated communication gaps as the foundation [46]. Communication gaps are pervasive challenges often identified in relation to ethnic-minority population groups [12, 46]. Cancer services could implement practices that empower consumers from ethnic minority backgrounds to identify their preferred communication strategy for when interacting with healthcare staff. Research from the disability sector indicates that providing options for different forms of communication (using a support person, using simple terminology, giving time to ask questions, speaking slowly) could enhance communication [54]. Providing options to the patient for indicating their preference may enhance cross-cultural communication. This will require adequate resources and change in processes at health service level and staff training for operationalisation.

Identifying patients from ethnic minority backgrounds is challenging and constrained by the availability and quality of health system administrative data. Data on language spoken at home were not reliably available across services. People from ethnic minority backgrounds in the Australian context were largely identified through country of birth data. In the Australian and international health system context, the need to collect a broader range of ethnicity data such as cultural heritage has been noted [12]. The EMR systems used by health services could allow for recording of the indicators in a way that they could be easily extracted. The process of recording data on language and ethnicity could be improved by designing a system in consultation with ethnic minority consumers [55] and the data recording completed with them. In the Australian health system context, the push towards the implementation of a single electronic medical record system (single digital patient record/SDPR) across public hospitals in a state of NSW may provide one such opportunity [56]. Co-designed collaborative development of a single EMR platform may also assist in addressing data inconsistencies (such as disparity in interpreter required category) across multiple different EMR systems.

### Limitations

For retrospective medical record reviews, study findings pertaining to the objectives are contingent on the quality of data collected and recorded in the notes. This has implications for the current study, where, due to lack of information or entry of data, some safety events may not have been included. Multisite medical record review studies are also subject to differences in findings (for example, rate of safety events was higher at Service C than other services) due to data collection and documentation practices at each site and type of EMR used. Different team members were also involved in data extraction, and this may have also shaped the study findings. We sought to overcome this limitation by standardising the data extraction process at each site, using a standardised data extraction tool, weekly meetings and training the researchers at the outset. Further, the clinician reviewers discussed their findings with each other and resolved any conflicts with discussion and input from third reviewer through weekly meetings as required. As ‘language spoken at home’ indicator was not routinely collected, this may mean that some eligible patient records would have been excluded. The study was undertaken at metropolitan sites, and further work is needed to examine the issue at regional and rural sites. Due to lower sample size, study may not be sufficiently powered to capture the differences for variables.

## Conclusion

This study provides novel evidence of the frequency and nature of patient safety events among patients from ethnic minority backgrounds in Australian cancer services. One in three patients accessing cancer care was exposed to at least one safety event. Medication-related safety events were of significant concern. More safety events occurred for patients that were reported to not require an interpreter. Communication gaps about care processes were commonly identified as a source of safety gaps in care. Strategies co-designed with end-users to enhance cross-cultural communication about care processes may provide a mechanism to improve safety in cancer care for ethnic minority population groups. Commitment from cancer services and executive leadership will be needed for implementation and evaluation of the co-designed strategies.

## Supplementary Information

Below is the link to the electronic supplementary material.

Supplementary file1 (DOCX 37 KB)

## Data Availability

Data can be requested by contacting the corresponding author (Dr Ashfaq Chauhan) or project lead (Professor Reema Harrison) and obtaining appropriate ethical approvals.
