# Supplementary material for: Patient Safety Events Among People from Ethnic Minority Backgrounds: A Retrospective Medical Record Review of Australian Cancer Services
Source: J Racial Ethn Health Disparities. 2025 Feb 27;13(2):1094–105. doi: 10.1007/s40615-025-02318-8 (PMC12966214; doi:10.1007/s40615-025-02318-8)
Supplement: Supplementary file 1 — Supplementary file1 (DOCX 37 KB) [file 40615_2025_2318_MOESM1_ESM.docx]

## Supplementary File 1: Oncology trigger tool^1^ adapted to Australian health system context.

| Code | Trigger | Australian Values |
| --- | --- | --- |
|  | Laboratory |  |
| L1 | Adrenal function studies | Same |
| L2 | Abnormal phosphate (> 5, < 1.5 mg/dL) | <0.75, >1.50 mmol/L  OR  18-20Y <0.75, >1.65mmol/L |
| L3 | Abnormal serum bicarbonate (< 18, > 36 mEq/L) | <22, >32 mmol/L |
| L4 | Abnormal serum bilirubin (> 2 mg/dL) | >21 umol/L |
| L5 | Abnormal serum calcium (> 12, < 7 mg/dL) | <2.10, >2.60 mmol/L |
| L6 | Abnormal serum magnesium (> 4, < 1.5 mg/dL) | <0.70, >1.10 mmol/L |
| L7 | Abnormal serum potassium (> 6, < 2.5 mEq/L) | <3.5, >5.2 mmol/L |
| L8 | Abnormal serum sodium (> 150, < 130 mEq/L) | <135, >145 mmol/L |
| L9 | Arterial blood gas (not in PACU/ICU) | Same |
| L10 | Bladder catheter and positive urine culture | Same |
| L11 | BNP (> 400 pg/mL) | >125 ng/L |
| L12 | *Clostridium difficile* toxin positive | Same |
| L13 | Elevated AST (> 300 units/L) or ALT (> 300 units/L) | >36 U/L (AST, ALT) |
| L14 | Elevated blood glucose (> 250 mg/dL) | <3.5, >5.4 mmol/L  OR  Fasting: >6.0  Non-Fasting: >7.7 |
| L15 | Elevated creatinine > 1 mg/dL and 50% greater than baseline | <45, >90 umol/L |
| L16 | Elevated INR (> 8) | Same |
| L17 | Elevated lipase (> 160 U/L) | 10 – 60 U/L |
| L18 | Elevated serum uric acid (> 10 mg/dL) | >1g/L  OR  M: >0.42mmol/L  F: >0.35mmol/L |
| L19 | Elevated troponin (> 0.64 ng/mL) | >14 ng/L |
| L20 | Elevated TSH (> 10 mcU/mL) | 12-19YR: >3.4mU/L  >19YR: >5.3mU/L |
| L21 | Low fibrinogen (< 100 mg/dL) | <1 g/L |
| L22 | Neutropenic fever (except in patients with leukemia or bone marrow transplant) | Same |
| L23 | Platelet count < 20,000 (except in patients with leukemia or bone marrow transplant) | Same  OR  <20 x 10^9^g/L |
| L24 | Positive blood culture without contaminant (eg, *Staphylococcus epidermidis*) | Same |
| L25 | Tylenol blood level^*^ | Paracetamol blood level |
|  | Orders |  |
| R1 | Acute inpatient dialysis | Same |
| R2 | Blood transfusion | Same |
| R3 | Cardiac defibrillator | Same |
| R4 | Chest x-ray in inpatient or urgent care center | Same (urgent care centre – Emergency Department) |
| R5 | Contact precautions/order for isolation | Same |
| R6 | Fistulogram/sinogram | Same |
| R7 | High-dose intravenous proton pump inhibitor (omeprazole, esomeprazole, and pantoprazole 80-mg bolus followed by 8 mg/h infusion) | Same |
| R8 | ICU transfer from floor | Same |
| R9 | Nasogastric tube (not in operating room) | Same |
| R10 | Noncontrast chest CT scan after radiation to the chest | Same |
| R11 | Percutaneous drain placement | Same/ Percutaneous drain insertion |
| R12 | Platelet transfusion (except in patients with leukemia or bone marrow transplant) | Same |
| R13 | Positive bone imaging test (plain films, CT scans) | Same |
| R14 | Positive lower-extremity ultrasound | Same |
| R15 | Positive upper-extremity ultrasound | Same |
| R16 | Rapid response team | PACE/RACE Call/ MET Call/Code Blue |
| R17 | Reintubation | Same |
| R18 | Steroid enema | Steroid enema (PREDSOL enema) |
| R19 | Use of pressors | Same |
|  | Consultations |  |
| C1 | Inpatient cardiology consult | Same |
| C2 | Inpatient gastroenterology consult | Same |
| C3 | Inpatient or outpatient IR consult (excluding referral for port placement) | Same |
| C4 | Inpatient surgery consults for nonsurgical patients | Same |
| C5 | Lymphedema consult | Same |
| C6 | Nephrology consult | Same |
| C7 | Neurology consult and noncontrast head CT scan | Same |
| C8 | Sitter and inpatient psychiatric consult | Same |
|  | General Care |  |
| G1 | Death in hospital | Same |
| G2 | Extravasation | Same |
| G3 | Fall | Same |
| G4 | Hospital readmission/urgent care visit within 72 h of hospital discharge or ambulatory surgery | Hospital readmission/emergency care visit within 72 hours of hospital discharge or ambulatory surgery |
| G5 | Low urine output (< 30 mL/h) | Same |
| G6 | Pressure ulcer | Same |
| G7 | Return to the operating room or IR within 30 days of surgery | Same |
|  | Vital Signs |  |
| V1 | Blood pressure (> 200/100 mmHg) | Same |
| V2 | Low oximetry results (Sao_2_ < 88%) | Same |
| V3 | Pain score (≥ 7) | Same |
| V4 | Temperature (< 35°C perioperatively) | Same |
|  | Medication related |  |
| M1 | Epinephrine | Same/Adrenaline |
| M2 | Flumazenil, glucagon, naloxone, protamine | SAME (Naloxone, BDL Flumazenil, Glucagen Hypokit, Protamine Sulfate) |
| M3 | Initiation of therapeutic anticoagulation | Same |
| M4 | Intravascular thrombolytic therapy | Same |
| M5 | Kayexalate | Sodium Polystyrene Sulfonate, Kayexalate, Resonium A |
| M6 | Methylnaltrexone | Same/ (Relistor) |
| M7 | Octreotide | Same |
| M8 | Oral anesthetics (eg, Magic Mouthwash, viscous lidocaine) | Same |
| M9 | Sodium thiosulfate, hyaluronidase, topical dimethylsulfoxide, dexrazoxane, phentolamine | Sodium thiosulfate, Hyaluronidase (Hyalase), Dimethylsulfoxide (DMSO), Dexrazoxane, Phentolamine (Regitine, Fentanor) |
| M10 | Total parenteral nutrition | Same |
| M11 | Use of intravenous glucose or dextrose | Same |
| M12 | Use of more than three doses of antiemetics within 24 h | Same |
| M13 | Vitamin K | Same |

^1^Lipitz-Snyderman A, Weingart SN, Anderson C, Epstein AS, Killen A, Classen D, Sima CS, Fortier E, Atoria CL, Pfister D, Lipitz-Snyderman A. ReCAP: Detection of potentially avoidable harm in oncology from patient medical records. Journal of oncology practice. 2016 Feb;12(2):178-9.)

## Supplementary File 2: Data collection tool

| **NUMBER** |  |
| --- | --- |
| **Date of Rv** |  |
| **Reviewer** |  |
| **UPI (Unique Patient Identifier)** |  |
| **Age at the time of first episode of care** |  |
| **Sex** |  |
| **Hospital code** |  |
| **Country of Birth** |  |
| **Language spoken at home** |  |
| **LSAH Code** | Non-English, English, Not available |
| **Preferred language** |  |
| **Preferred language Code** | Non-English, English, Not available |
| **Interpreter required** | Yes, No |
| **Diagnosis type** |  |
| **Date of Diagnosis** |  |
| **Type of treatment/s received** | Surgery, Chemo, Radiation, Surgery + Chemo, Surgery + Radiation, Chemo + Radiation, Surgery + Chemo + Radiation, Other |
| **Did patient receive palliative care treatment over 1 year period** | Yes, No |
| **Date when first episode of care commenced at the participating sites** |  |
| **Was Trigger identified** | Yes, No |
| **What trigger was identified - OTT** |  |
| **No of presentation to ED in a one year follow up since first episode of care** |  |
| **Did safety event occur** | Yes, No |
| **Date of safety event reported or noted** |  |
| **NUMBER OF SAFETY EVENTS RECORDED IN 1 YEAR FOLLOW UP PERIOD** |  |
| **Was interpreter used for the episodes of care when safety event occurred** | Interpreter used, Interpreter not available, Family used as interpreter, Interpreter not required, Information not available, Not applicable |
| **Describe the safety event identified** |  |
| **Communication was made about the safety event with consumer?** | Yes, No, Information not available, Not applicable, Patient identified |
| **Delivery of care mode when safety event occurred.** | Inpatient, Outpatient, ED |
| **What harm occurred - narrative description** |  |
| **Incident Type as per WHO taxonomy** | (All listed plus Not applicable) |
| **Narrative description relating to preventability of harm/or error** |  |
| **General Noteworthy Observations** |  |

## Supplementary File 3: List of Electronic Medical Record (EMR) systems used across the four services.

| **Service** | **Inpatient EMR System** | **Outpatient EMR System** |
| --- | --- | --- |
| Service A (NSW) | Cerner Power Chart | Mosaiq |
| Service B (NSW) | Cerner Power Chart | Aria |
| Service C (VIC) | Cerner Power Chart  SMR (Scanned Medical Record) | Mosaiq  SMR (Scanned Medical Record) |
| Service D (VIC) | BOSSNet DMR  Cerner Power Chart | BOSSNet DMR  Cerner Power Chart |

## 
